# Supplementary material for: Stromal Cell Subsets Modulate T-cell Infiltration in Early Breast Cancer
Source: Cancer Res Commun. 2026 Jul 8;6(7):1605–18. doi: 10.1158/2767-9764.CRC-25-0709 (PMC13343345; doi:10.1158/2767-9764.CRC-25-0709)

**Supplementary Figure 1.** Association of CD8 T cell percentages with clinical characteristics in the luminal cohort. Clinical variables include age at diagnosis (**A**), node metastasis (**B**), tumour grade (**C**), molecular subtypes (**D**), and tumour size (**E**).


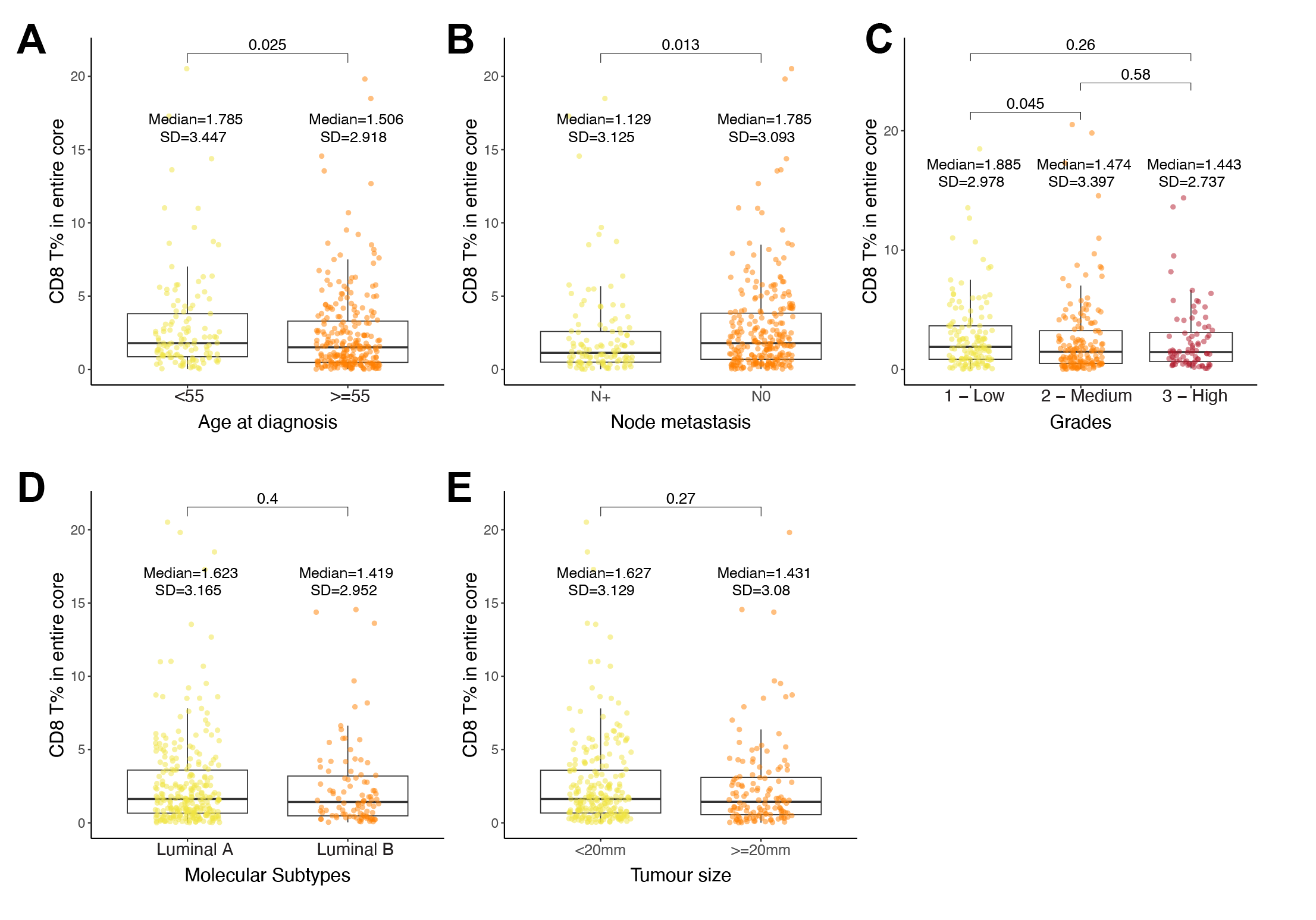

Supplement: Supplementary Figure 1 — Association of CD8 T cell percentages with clinical characteristics in the luminal cohort. [file crc-25-0709_supplementary_figure_1_suppsf1.docx]
