# Supplementary material for: Stromal Cell Subsets Modulate T-cell Infiltration in Early Breast Cancer
Source: Cancer Res Commun. 2026 Jul 8;6(7):1605–18. doi: 10.1158/2767-9764.CRC-25-0709 (PMC13343345; doi:10.1158/2767-9764.CRC-25-0709)

**Supplementary Figure 2. A.** Percentage of PVLs with endothelial cells present within a 10–100 µm radius in the TNBC cohort. **B, C**. Correlation between the percentage of distal endothelial cells and distal PVLs in luminal (**B**) and TNBC (**C**) cohorts. Disseminated endothelial cells are defined as those without a PVL within 100 µm.


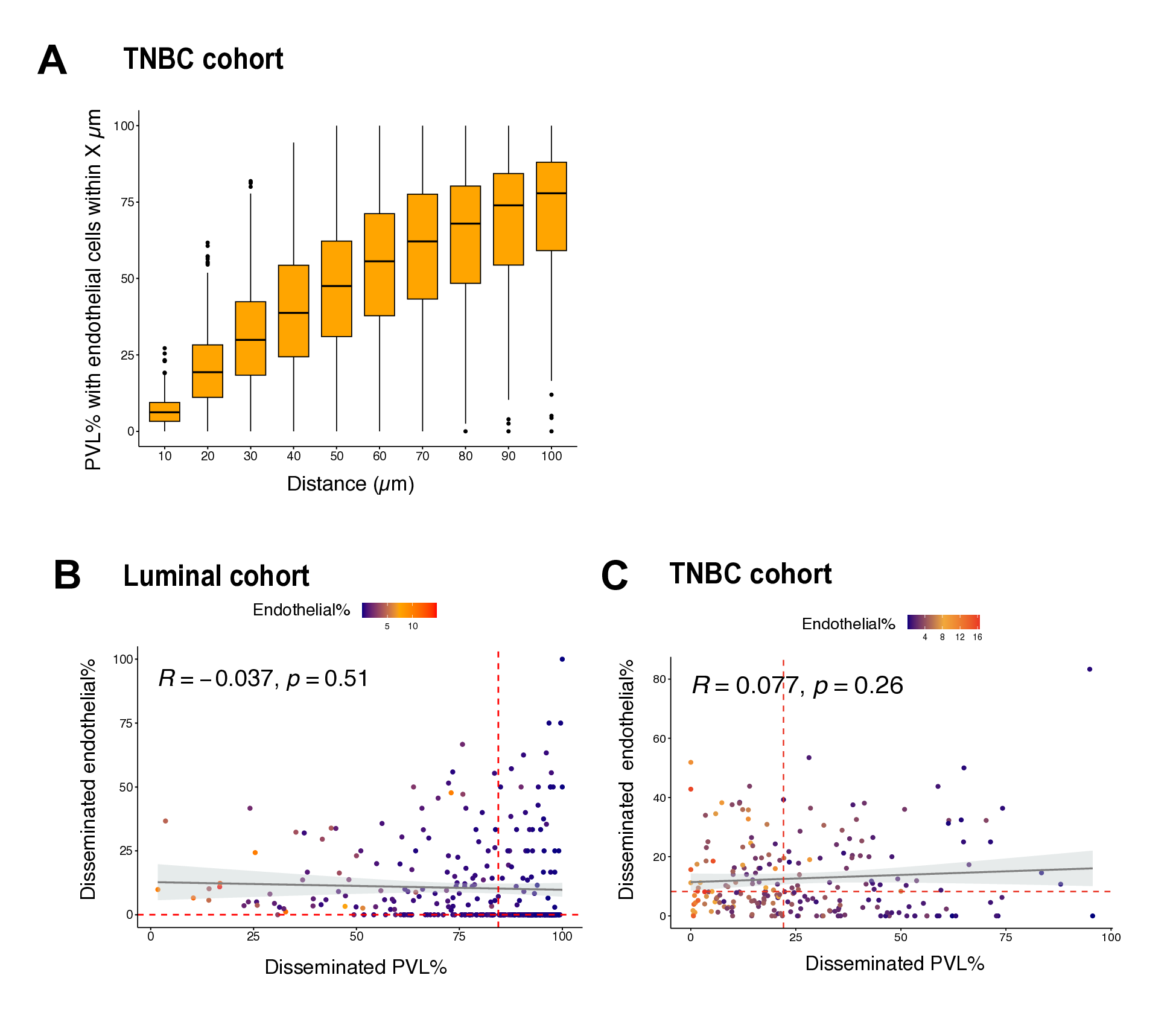

Supplement: Supplementary Figure 2 — Correlation between PVLs and endothelial cells. [file crc-25-0709_supplementary_figure_2_suppsf2.docx]
