# Supplementary material for: Stromal Cell Subsets Modulate T-cell Infiltration in Early Breast Cancer
Source: Cancer Res Commun. 2026 Jul 8;6(7):1605–18. doi: 10.1158/2767-9764.CRC-25-0709 (PMC13343345; doi:10.1158/2767-9764.CRC-25-0709)

**Supplementary Figure 3.** **A.** KM curves for OS stratified by the percentage of PD1⁻CD8⁺ T cells in myCAF-high vs. myCAF-low groups. **B.** Multivariable Cox proportional hazard regression analysis of OS for the proportion of PD1⁻CD8⁺ T cells% in myCAF-low group, considering age, lymph node metastasis, chemotherapy and tumour size.


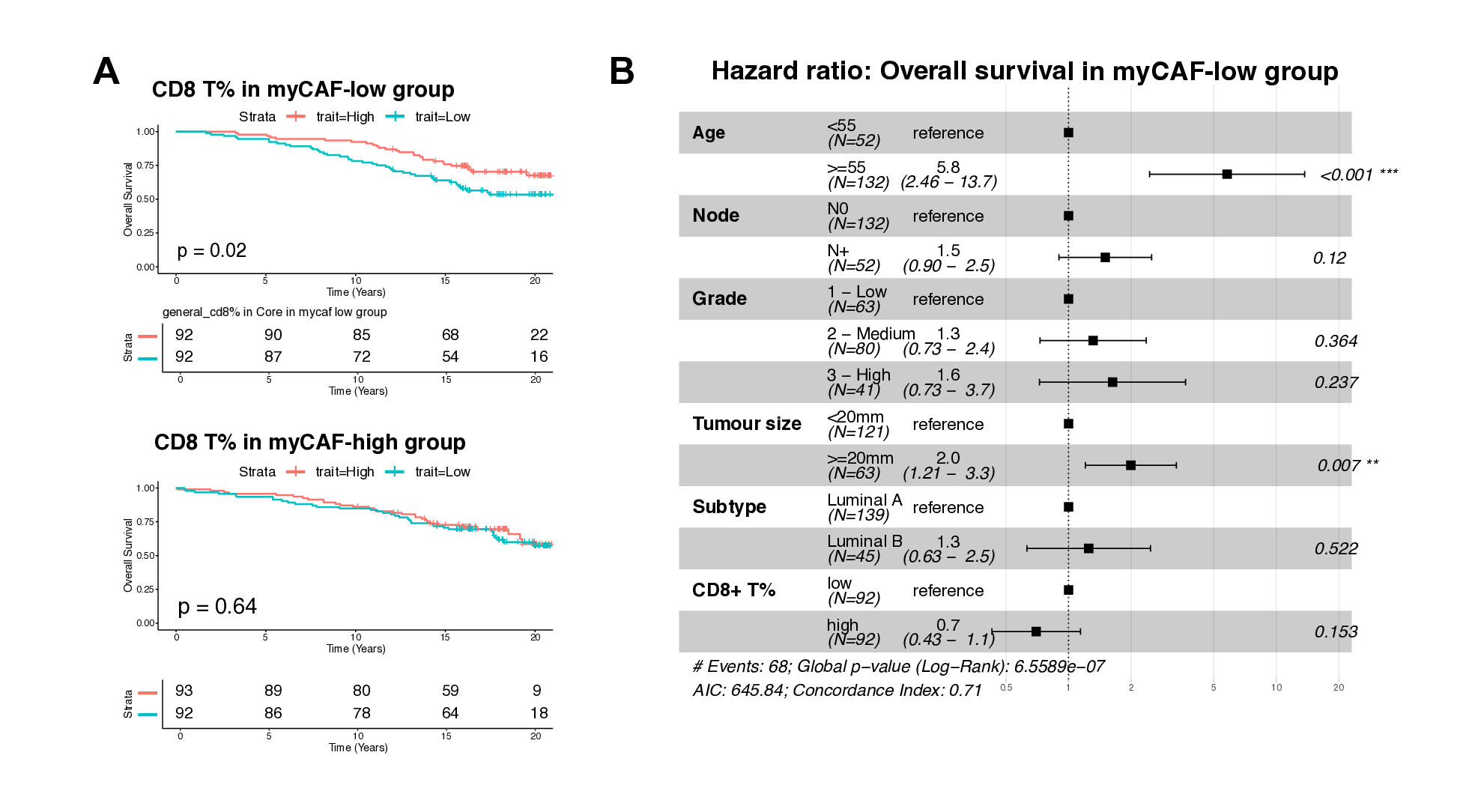

Supplement: Supplementary Figure 3 — Survival analyses of PD1−CD8+ T cells in myCAF-high vs. myCAF-low groups [file crc-25-0709_supplementary_figure_3_suppsf3.docx]
