# Supplementary material for: Stromal Cell Subsets Modulate T-cell Infiltration in Early Breast Cancer
Source: Cancer Res Commun. 2026 Jul 8;6(7):1605–18. doi: 10.1158/2767-9764.CRC-25-0709 (PMC13343345; doi:10.1158/2767-9764.CRC-25-0709)

**Supplementary Figure 4. A.** Association of myCAF-related spatial features with clinical variables in the luminal cohort. Patients were stratified by clinical variables. Dot colors indicate patient groups in which the feature value is increased. Dot outlines denote statistical significance of feature enrichment (red: significant; grey: not significant), assessed by the Wilcoxon rank-sum test with p-values corrected using the Benjamini-Hochberg method. **B.** Endothelial cell percentages in the stromal region across tumours of 1 to 3 grades. **C.** Association of myCAF-related spatial featur es with clinical variables in the TNBC cohort.


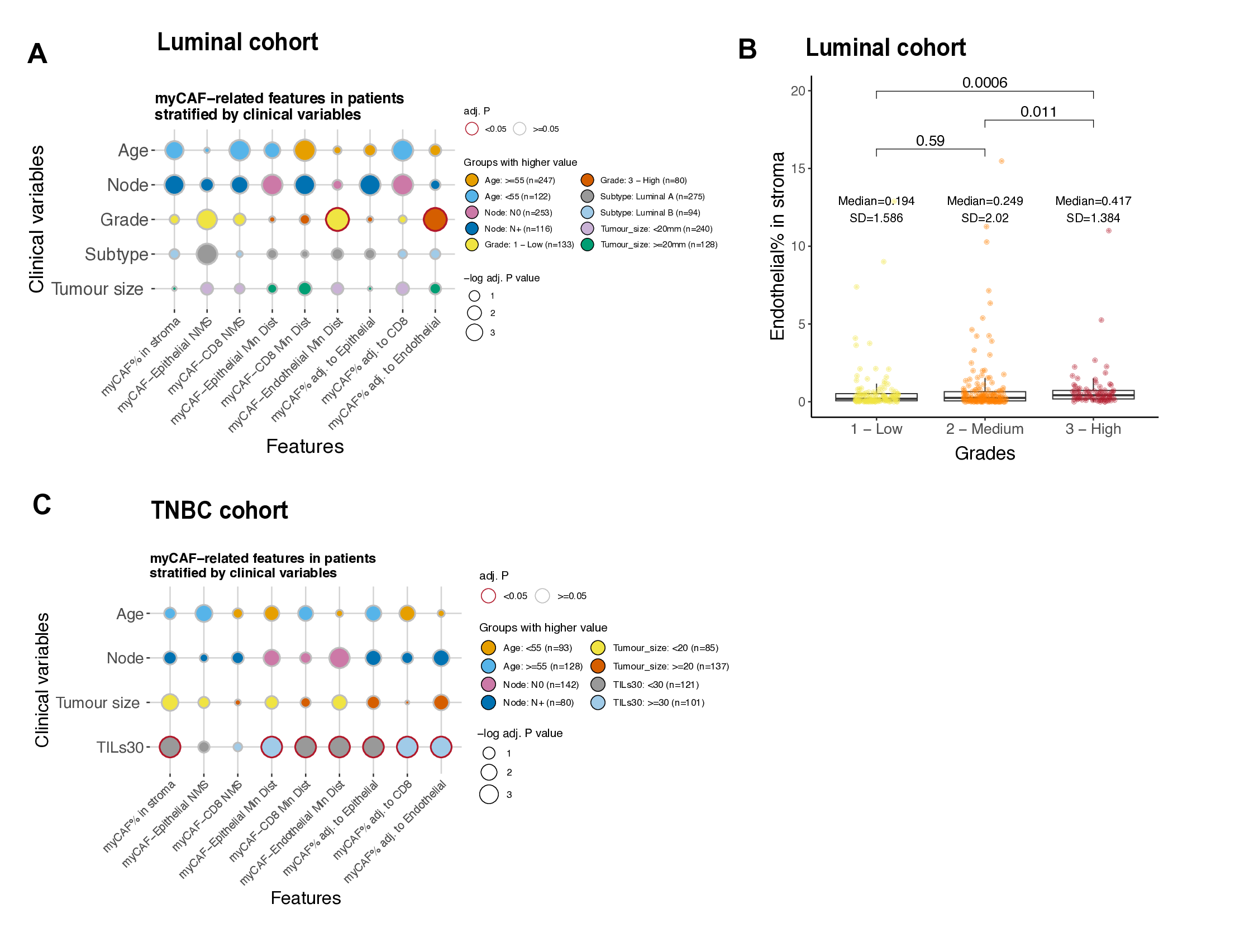

Supplement: Supplementary Figure 4 — Association of myCAF-related spatial features with clinical variables. [file crc-25-0709_supplementary_figure_4_suppsf4.docx]
