# Supplementary material for: Stromal Cell Subsets Modulate T-cell Infiltration in Early Breast Cancer
Source: Cancer Res Commun. 2026 Jul 8;6(7):1605–18. doi: 10.1158/2767-9764.CRC-25-0709 (PMC13343345; doi:10.1158/2767-9764.CRC-25-0709)
Supplement: Supplementary Table 1 — Antibodies used in mIF panel. [file crc-25-0709_supplementary_table_1_suppst1.docx]

**Supplementary table 1.** Antibodies used in mIF panel.

| **Antibody** | **Manufacturer and catalogue number** | **Epitope** | **Dilution** |
| --- | --- | --- | --- |
| panCK | Abcam ab27988 | OPAL650 | 1: 2000 |
| PDGFRβ (CD140b) | Abcam ab32570 | OPAL 540 | 1: 1000 |
| aSMA | Abcam ab5694 | OPAL780 | 1: 500 |
| CD146 | Abcam ab75769 | OPAL 570 | 1: 1250 |
| THY1(CD90) | Abcam ab133350 | OPAL 690 | 1: 4000 |
| CD8 | Invitrogen MA5-13473 | OPAL 620 | 1: 1000 |
| PD-1 | Abcam ab137132 | OPAL 520 | 1: 50 |
| CD31 | Agilent Technologies/DAKO M0823 | OPAL 480 | 1: 100 |
