# Supplementary material for: Stromal Cell Subsets Modulate T-cell Infiltration in Early Breast Cancer
Source: Cancer Res Commun. 2026 Jul 8;6(7):1605–18. doi: 10.1158/2767-9764.CRC-25-0709 (PMC13343345; doi:10.1158/2767-9764.CRC-25-0709)
Supplement: Supplementary Table 2 — Marker combinations for defining cell types in the mIF panel. [file crc-25-0709_supplementary_table_2_suppst2.docx]

**Supplementary table 2.** Marker combinations for defining cell types in the mIF panel. Alternative combinations were separated by semi comma.

| **Cell type** | **Phenotype marker combinations** |
| --- | --- |
| myCAF | a-SMA+CD140b+ |
| Epithelial cells | PanCK+; CD31+PanCK+ |
| imPVL | CD146+Thy1+; CD140b+CD146+Thy1+ |
| dPVL | CD146+; CD140b+CD146; CD140b+CD146+a-SMA+ |
| Endothelial cells | CD31+; CD31+CD146+; CD31+CD140b+ |
| iCAF | CD140b+ |
| PD1-CD8+ T cells | CD8+; CD8+CD140b+; CD8+PanCK+ |
| PD1+CD8+ T cells | PD1+CD8+; PD1+CD8+CD140b+; PD1+CD8+PanCK+ |
