# Supplementary material for: Stromal Cell Subsets Modulate T-cell Infiltration in Early Breast Cancer
Source: Cancer Res Commun. 2026 Jul 8;6(7):1605–18. doi: 10.1158/2767-9764.CRC-25-0709 (PMC13343345; doi:10.1158/2767-9764.CRC-25-0709)
Supplement: Supplementary Table 3 — Description of features selected for assessing the association with clinicopathological variables. [file crc-25-0709_supplementary_table_3_suppst3.docx]

**Supplementary table 3.** Description of features selected for assessing the association with clinicopathological variables.

| **Features** | **Description** |
| --- | --- |
| % in stroma | Proportions of CAF subsets among all detected cells in the stromal region |
| Epithelial_NMS | Normalized mixing score between CAF subsets and epithelial cells |
| CD8_NMS | Normalized mixing score between CAF subsets and CD8 T cells |
| Endothelial_NMS | Normalized mixing score between CAF subsets and endothelial cells |
| Epithelial Min Dist | Averaged distance from CAF subsets to the nearest epithelial cell |
| CD8 Min Dist | Averaged distance from CAF subsets to the nearest CD8 T cells |
| Endothelial Min Dist | Averaged distance from CAF subsets to the nearest endothelial cells |
| adj. to Epithelial | Proportions of CAF subsets located within 30µm radius to epithelial cells |
| adj. to CD8 | Proportions of CAF subsets located within 30µm radius to CD8 T cells |
| adj. to Endothelial | Proportions of CAF subsets located within 30µm radius to endothelial cells |
