# Supplementary material for: Stromal Cell Subsets Modulate T-cell Infiltration in Early Breast Cancer
Source: Cancer Res Commun. 2026 Jul 8;6(7):1605–18. doi: 10.1158/2767-9764.CRC-25-0709 (PMC13343345; doi:10.1158/2767-9764.CRC-25-0709)
Supplement: Supplementary Table 4 — Xenium gene list for custom-designed add-ons [file crc-25-0709_supplementary_table_4_suppst4.docx]

**Supplementary table 4.** Xenium gene list for custom-designed add-ons

| AC244453.1 | ACKR1 | ACTA2 | ALDH1A1 | ANKRD22 | ATP8B1 | C5AR2 | CCL21 | CD69 | CD74 |
| --- | --- | --- | --- | --- | --- | --- | --- | --- | --- |
| CDH19 | CHI3L1 | CISH | CLEC4E | CNN1 | COL15A1 | CPA3 | CUEDC1 | CXCL14 | CXCL8 |
| CYB561 | DAPK2 | DCN | DOCK1 | DPM2 | EBAG9 | EGLN2 | ELF3 | ELL3 | EPS15L1 |
| FBLN1 | FGF18 | FKBP4 | FKBP5 | FLOT1 | FN1 | GFPT2 | GNB1 | GREB1 | GRIP1 |
| HDAC11 | HHIP | HLA-DRA | HLA-DRB1 | HPN | HSPB1 | IGFBP4 | IGFL2 | IRF9 | ITGA10 |
| KDM4C | KLF10 | KLF2 | KRT15 | KRT19 | KRT5 | KRT8 | LGI4 | LUM | LYVE1 |
| LYZ | MEF2B | MEG8 | MEOX2 | MMP1 | MMP2 | MNDA | MTRNR2L12 | MYH11 | NID2 |
| NTAN1 | OGN | OSMR | P4HA2 | PARD6G | PARP9 | PCOLCE | PLA2G2A | PLP1 | PTPRC |
| PTPRD | RAMP3 | RASGEF1B | RGS13 | S100A8 | S100A9 | SCD | SFRP2 | SGK3 | SLC14A1 |
| SPDEF | SPNS2 | SPP1 | SRGN | SRSF5 | TAGLN | TPSB2 | UBE2B | VIM | VWF |
