# Supplementary material for: Stromal Cell Subsets Modulate T-cell Infiltration in Early Breast Cancer
Source: Cancer Res Commun. 2026 Jul 8;6(7):1605–18. doi: 10.1158/2767-9764.CRC-25-0709 (PMC13343345; doi:10.1158/2767-9764.CRC-25-0709)
Supplement: Supplementary Table 5 — Marker gene combinations for defining cell types in Xenium. [file crc-25-0709_supplementary_table_5_suppst5.docx]

**Supplementary table 5.** Marker gene combinations for defining cell types in Xenium. Alternative combinations were separated by semi comma.

| **Cell type** | **Phenotype marker combinations** |
| --- | --- |
| myCAF | *ACTA2*+*PDGFRB*+ |
| Epithelial cells | *EPCAM*+; *PECAM1*+*EPCAM*+ |
| imPVL | *MCAM*+*THY1*+; *MCAM*+*THY1*+*PDGFRB*+ |
| dPVL | *MCAM*+; *PDGFRB*+*MCAM*+; *PDGFRB*+*MCAM*+*ACTA2*+ |
| Endothelial cells | *PECAM1*+; *PECAM1*+*MCAM*+; *PECAM1*+*PDGFRB*+ |
| iCAF | *PDGFRB*+ |
| PD1-CD8+ T cells | *CD8A*+; *CD8A*+*PDGFRB*; *CD8A*+*EPCAM*+ |
| PD1+CD8+ T cells | *PDCD1*+*CD8A*+; *PDCD1*+*CD8A*+*PDGFRB*+; *PDCD*+*CD8A*+*EPCAM*+ |
