# Supplementary material for: Stromal Cell Subsets Modulate T-cell Infiltration in Early Breast Cancer
Source: Cancer Res Commun. 2026 Jul 8;6(7):1605–18. doi: 10.1158/2767-9764.CRC-25-0709 (PMC13343345; doi:10.1158/2767-9764.CRC-25-0709)
Supplement: Supplementary Table 6 — Univariate and multivariate Cox regression of clinicopathological predictors of overall survival in in the luminal cohort. [file crc-25-0709_supplementary_table_6_suppsts6.docx]

**Supplementary table 6:** Univariate and multivariate Cox regression of clinicopathological predictors of overall survival in in the luminal cohort. p<0.05 was considered statistically significant (HR: hazard ratio; CI: confidence interval).

|  | **Univariate analysis** | | | **Multivariate analysis** | | |
| --- | --- | --- | --- | --- | --- | --- |
|  | HR | 95% CI | P-value | HR | 95% CI | P-value |
| Age (>=55) | 3.12 | 1.95-4.97 | **<0.001** | 3.55 | 2.21-5.71 | **<0.001** |
| Node (N^+^) | 1.55 | 1.09-2.19 | **0.014** | 1.40 | 0.97-2.02 | 0.072 |
| Grade (2-Medium) | 1.58 | 1.06-2.35 | **0.023** | 1.35 | 0.89-2.04 | 0.16 |
| Grade (3-High) | 1.35 | 0.84-2.18 | 0.217 |  |  |  |
| Chemo (No) | 1.32 | 0.79-2.19 | 0.289 |  |  |  |
| Boost (No) | 1.36 | 0.96-1.91 | 0.080 |  |  |  |
| Tumour size (>=20mm) | 1.46 | 1.03-2.06 | **0.032** | 1.41 | 0.97-2.05 | 0.072 |
| Molecular subtype (luminal B) | 1.86 | 1.30-2.66 | **<0.001** | 2.07 | 1.38-3.12 | **<0.001** |
