# Supplementary material for: Stromal Cell Subsets Modulate T-cell Infiltration in Early Breast Cancer
Source: Cancer Res Commun. 2026 Jul 8;6(7):1605–18. doi: 10.1158/2767-9764.CRC-25-0709 (PMC13343345; doi:10.1158/2767-9764.CRC-25-0709)
Supplement: Supplementary Table 7 — Univariate and multivariate Cox regression of clinicopathological predictors of overall survival in in the TNBC cohort. [file crc-25-0709_supplementary_table_7_suppst7.docx]

**Supplementary table 7.** Univariate and multivariate Cox regression of clinicopathological predictors of overall survival in in the TNBC cohort. p<0.05 was considered statistically significant (HR: hazard ratio; CI: confidence interval).

|  | **Univariate analysis** | | | **Multivariate analysis** | | |
| --- | --- | --- | --- | --- | --- | --- |
|  | HR | 95% CI | P-value | HR | 95% CI | P-value |
| Age (>=55) | 2.77 | 1.59-4.84 | **<0.001** | 2.75 | 1.48-5.10 | **0.0014** |
| Node (N^+^) | 2.39 | 1.47-3.91 | **<0.001** | 2.80 | 1.56-5.02 | **<0.001** |
| Chemo (no) | 2.07 | 1.23-3.47 | **0.006** | 1.86 | 1.03-3.36 | **0.039** |
| Tumour size (>=20mm) | 2.03 | 1.17-3.54 | **0.012** | 1.66 | 0.92-3.01 | 0.092 |
| TILs30 (>=30) | 0.58 | 0.35-0.97 | **0.037** | 0.56 | 0.33-0.96 | **0.036** |
